# The relationships among perceived league product quality, spectator satisfaction, trust, team identification, and loyalty in the Chinese Super League

**DOI:** 10.1371/journal.pone.0350647

**Published:** 2026-06-08

**Authors:** Fei Liu, Sisi Wu, Jingyin Zhou, Mu Fan, Fengqin Tian

**Affiliations:** 1 Football School, Guangzhou Sport University, Guangzhou, China; 2 Department of Sport Science, Zhejiang University, Hangzhou, China; 3 Sports Management School, Guangdong Vocational Institute of Sport, Guangzhou, China; Government Law College, INDIA

## Abstract

**Objective:**

This study integrates perceived league product quality, spectator satisfaction, trust, team identification, and loyalty into a unified analytical framework to examine their relationships, thereby providing empirical evidence to inform more effective marketing strategies for the Chinese Super League (CSL).

**Methods:**

Guided by the ABC attitude model and conceptual definitions, as well as prior research, the study develops a theoretical model and an initial pool of measurement items. Expert consultation and a pilot study were subsequently conducted to refine items. On-site questionnaires were administered to the CSL spectators, yielding 320 responses, of which 278 were valid. Data were analyzed using SPSS 22.0, AMOS 23.0, and PROCESS v4.2 to assess scale reliability and validity, evaluate the structural model fit, and test path coefficients and mediation effects.

**Results:**

The theoretical model demonstrated a good fit to data, path analysis revealed that perceived league product quality had significant positive effects on satisfaction (β = 0.759, *p* < 0.01), trust (β = 0.540, *p* < 0.01), and team identification (β = 0.570, *p* < 0.01), but not on loyalty (β = 0.205, *p* > 0.05). Satisfaction (β = 0.038, *p* > 0.05) and trust (β = 0.029, *p* > 0.05) did not significantly affect loyalty, whereas team identification had a significant positive effect on loyalty (β = 0.534, *p* < 0.01). Mediation analysis indicated that the indirect effects of league product quality on loyalty, mediated by satisfaction and trust, were not significant, whereas the indirect effect through team identification was significant. Furthermore, a chain mediation pathway of league product quality → Satisfaction → Team Identification → Loyalty was found to be statistically significant.

**Conclusion:**

Higher perceived league product quality significantly enhances spectator satisfaction, trust, and team identification. Among these factors, team identification emerges as the strongest predictor of spectator loyalty and serves as the primary mediating mechanism through which perceived league product quality is translated into sustained spectator loyalty.

## Introduction

High-level professional leagues play an important role in driving both economic growth and social progress. For example, during the 2019/20 England Premier League (EPL) season, the average stadium attendance exceeded 40,000 for the first time, and the stadium utilization rate reached a record 98.7%. The league generated approximately £3.6 billion in tax revenue and created about 94,000 jobs for the United Kingdom [1]. The Chinese Super League (CSL), established in 2004 and modeled after the EPL, is the highest-tier professional football league in China. However, CSL and its clubs have long faced challenges, including a weak public image, relatively low brand value, and persistent financial losses. The combined losses of the 16 CSL clubs reached RMB 4.8 billion in the 2017 season [2], and the average attendance in the 2025 season was 25,754 per match [3]. Therefore, the National Development and Reform Commission issued the *Medium to Long-Term Development Plan for Chinese Football (2016–2050)*, which emphasizes expanding CSL’s influence, enhancing its brand value, and increasing average attendance to a world-class level.

With the globalization and commercialization of sports, professional sports organizations and clubs face increasingly intense competition in both domestic and international markets. From a marketing perspective, professional sports leagues are essentially entertainment products jointly produced by teams, primarily aimed at delivering engaging viewing experiences for spectators (who are also customers) while generating commercial and social value for broadcasters, sponsors, and other stakeholders. Governing bodies and clubs must adopt a spectator-oriented approach, prioritizing both the breadth and quality of their product offerings.

Attitude refers to the sum of an individual’s thoughts, feelings, and beliefs concerning an attitude object—such as an entity, person, or event [4]. It comprises three interrelated components: cognition, affect, and behavior [5]. As the CSL enters a transitional phase described as the “post-splurge era”, spectators’ attitudes towards league products have become increasingly important in shaping the CSL’s brand image, commercial value, and long-term sustainability. Existing research has largely examined isolated attitudinal dimensions, such as CSL brand perception [6], the formation of fan behavioral loyalty [7], and spectators’ consumption demand [8]. However, few studies have analyzed the complex relationships among spectators’ cognitive, affective, and behavioral variables within an integrated framework.

The ABC attitude model (Affection–Behavior–Cognition) provides a well-established framework for analyzing consumer attitudes [9]. Building on this model, this study employs perceived league product quality as a cognitive variable; spectator satisfaction, trust, and team identification as affective variables; and behavioral loyalty as the behavioral outcome. By examining the structural relationships among these variables, this study aims to provide empirical evidence to support more effective marketing strategies for the CSL governing authorities and clubs.

## Theoretical frameworks

### ABC attitude model

The ABC attitude model originated from empirical research on attitude structure in social psychology in the mid-twentieth century. The work of Rosenberg and Hovland (1960) contributed to the systematic development of the ABC model [10]. Cognition refers to consumers’ evaluations, beliefs, or perceptions of an object after it has been exposed to external stimuli. Affection reflects consumers’ emotional responses or feelings generated from their overall evaluation of a product or service. Behavior represents consumers’ tendencies or intentions to engage in certain actions toward that object [11,12]. Due to its conceptual clarity, the ABC model has been widely applied across various research domains such as public policy and consumer decision-making [13,14].

Within the context of professional sport, perceived product quality represents the cognitive foundation of spectators’ attitudes toward league offerings. Affective responses typically include satisfaction, trust, and team identification, which capture spectators’ emotional reactions, confidence in the organization, and psychological attachment to teams. Behavioral outcomes are commonly reflected in loyalty behaviors, such as repeat attendance, continued support, and positive word-of-mouth.

In this study, although league product quality is conceptualized at the league level, professional leagues inherently operate as nested systems in which clubs function as constituent units that both compete and collaborate. The league product, encompassing both core and peripheral attributes, is primarily delivered through clubs. Therefore, spectators’ perceptions of league product quality are largely formed from their evaluations of these club-delivered elements. It is theoretically reasonable to expect that perceived league product quality influences club-level psychological and behavioral outcomes.

### The concepts

#### League product quality.

Product quality refers to the degree of excellence or inferiority inherent in a product. In this study, both core product quality and peripheral product quality are examined from the spectator’s perspective. Scholars generally agree that spectator sports products have two key components: core and peripheral products [15,16]. The core product primarily is mainly the match itself and includes two major components: (1) Team characteristics, such as win–loss records, team history, the number of star players, and the quality of the opposing teams. (2) On-field player performance, including skills, tactics, teamwork, and overall effort [17,18]. Some studies also include attributes like match scheduling, league-assigned home region, competition formats (e.g., playoffs), outcome uncertainty, and the entertainment value of matches [19]. The peripheral product serves as a supplement to the core product. It includes features such as food and beverage services, fan merchandise sales, halftime entertainment, audiovisual displays, stadium facilities, and accessibility to the venue [20].

#### Spectator satisfaction.

Customer satisfaction is a post-purchase emotional reaction that compares the actual performance of a product/service against pre-purchase expectations, often resulting in feelings of pleasure or fulfillment [21]. In sport management, spectator satisfaction refers to pleasurable and favorable responses to a sporting game and the ancillary services provided during the match [22,23].

#### Spectator trust.

In organizational behavior, trust describes the confidence one has in the actions or intentions of an organization or group, rooted in the expectation that the organization will act ethically, fairly, and benevolently, while respecting the rights of others [24]. Trust is present when one party believes in the reliability and integrity of its counterpart [25]. In sport consumption, spectators may extend trust to sport organizations in ways analogous to interpersonal trust, and expect consistency, reliability, and goodwill from organizational entities [26].

#### Team identification.

In sports studies, team identification refers to the degree of psychological connection an individual feels with a sports entity or team. It emerges when individuals develop the cognition that they support and follow a particular team [27]. This identification is characterized by a strong psychological and emotional attachment that becomes integrated into one’s self-concept, causing team successes and failures to be personally experienced and fostering a sense of belonging and shared identity with fellow fans.

#### Spectator loyalty.

In consumer research, behavioral loyalty refers to the sustained and repetitive choice and support demonstrated by consumers or users in their actual actions, based on a positive attitude toward a specific brand, product, service, or organization. Behavioral loyalty refers to consumers’ (future) behaviors or behavioral intentions. In a sports context, behavioral loyalty may include renewing or extending club memberships, increasing membership scope, recommending the club to others, and other repeat-consumption behaviors [28,29].

### Hypothesis

**Fig 1:**
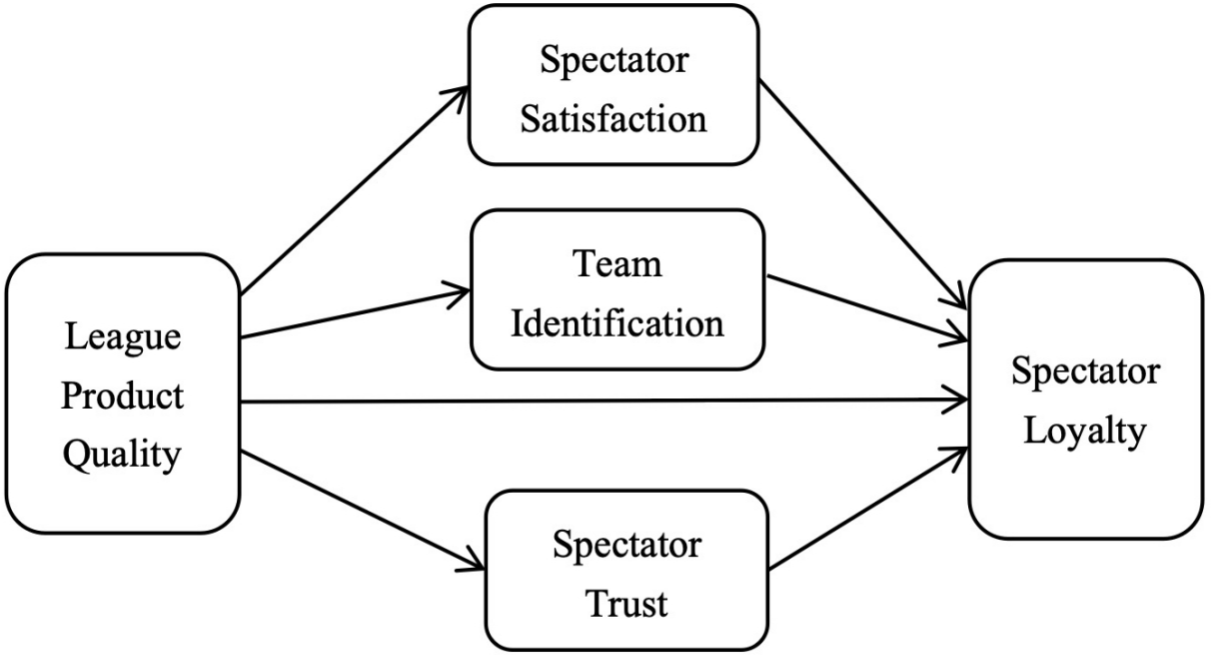
Theoretical model.

Within the ABC attitude model, cognition serves as the foundation of both affect and behavior, affect functions as a mediator between cognition and behavior, and behavior represents the outcome of cognitive and affective processes [30]. Based on this theoretical logic, a theoretical model is developed (Fig 1), and the following hypotheses are proposed.

Cognitive → Affective Relationships

H1: Perceived league product quality has a positive effect on spectator satisfaction.

H2: Perceived league product quality has a positive effect on spectator trust.

H3: Perceived league product quality has a positive effect on team identification.

Affective → Behavioral Relationships

H4: Spectator satisfaction has a positive effect on loyalty.

H5: Spectator trust has a positive effect on loyalty.

H6: Team identification has a positive effect on loyalty.

Cognitive → Behavioral Relationship

H7: Perceived league product quality has a positive effect on loyalty.

Mediation Hypotheses

H8a: Spectator satisfaction mediates the relationship between perceived league product quality and loyalty.

H8b: Spectator trust mediates the relationship between perceived league product quality and loyalty.

H8c: Team identification mediates the relationship between perceived league product quality and loyalty.

## Methods

### Instruments

Based on the conceptual definitions and prior studies, an initial item pool of the scale was developed. The league product quality scale consisted of two subscales (core product quality and peripheral product quality), comprising a total of 38 items. Spectator satisfaction, trust, team identification, and loyalty were measured using 4, 3, 5, and 5 items, respectively. Subsequently, six football experts were invited to evaluate the content validity. Among them, one was an international-level football referee, two were CSL match supervisors, and the other three were professional football researchers. Based on their feedback, two items, “rationality of league policies” in core product quality and “adequacy of security measures” in peripheral product quality, were deleted. An online pilot survey was distributed to football students at a sports university and members of a football fan club. The inclusion criterion required respondents to have attended a CSL match in person. The recruitment period began on 06/06/2025 and ended on 10/06/2025. A total of 142 questionnaires were collected, and after excluding invalid responses, 87 valid questionnaires remained.

To refine the items, two methods were used: (1) High—low group comparison. Respondents were ranked by total scores, with the top 27% forming the high-score group and the bottom 27% forming the low-score group; and (2) Item-total correlation test. If an item’s correlation coefficient with the total score of the scale was below 0.4, it indicated a weak association with the overall construct and was considered for deletion [31]. As a result, the item “current league ranking of the home club” (a correlation coefficient of 0.288) in the core product quality subscale was deleted. All remaining items exhibited correlation coefficients above 0.40 and significant differences between high- and low-score groups (*p* < 0.01).

Following these procedures, the final measurement scale was established. Table 1 presents the literature sources for all items. The league product quality uses a 7-point Likert scale ranging from 1 (very poor) to 7 (very good). Spectator satisfaction, trust, team identification, and loyalty use a 7-point Likert scale ranging from 1 (Strongly disagree) to 7 (Strongly agree).

**Table 1:** Items description and its literature sources.

Variables	Items	Literature Sources
**League Core Product Quality** **(LCPQ)**	Historical prestige of the home team (CSQ1)	Byon et al. (2013) [16]; Sarstedt et al. (2014) [32]; Warren (2011) [33]; Yoshida and James (2010) [22]; Robinson et al. (2012) [34]; National Football League (2025) [35]; Murray (2018) [36]; Andreff (2019) [37]; Zglinski (2022) [38]
	Number of star players in the home team (CSQ2)	
	Overall technical and tactical ability of foreign players in the home team (CSQ3)	
	Overall technical and tactical ability of domestic players in the home team (CSQ4)	
	Reputation of the home team head coach (CSQ5)	
	Historical prestige of the away team (CSQ6)	
	Number of star players in the away team (CSQ7)	
	Overall technical and tactical ability of foreign players in the away team (CSQ8)	
	Overall technical and tactical ability of domestic players in the away team (CSQ9)	
	Reputation of the away team’s head coach（CSQ10）	
	Competitive balance between the two teams (CSQ11)	
	Effort and dedication of players during the match (CSQ12)	
	Compliance of players’ behavior during the match (CSQ13)	
	Overall refereeing quality (CSQ14)	
	Reasonableness of the league match schedule (CSQ15)	
	Stability of the league match schedule (CSQ16)	
	Stability of the league system (e.g., promotion and relegation rules) (CSQ17)	
**League Peripheral Product Quality** **(LPPQ)**	Visual appeal of stadium design (PSQ1)	Yoshida and James (2010) [22]; Sarstedt et al. (2014) [32]; Wakefield et al. (1996) [39]; Jang et al. (2020) [40]; Byon et al. (2013) [16]; Höck et al. (2010) [41]; Uhrich and Benkenstein (2010) [42]; Veeraraghavan and Vaidyanathan (2012) [43]; Deloitte (2019) [44]; Hill and Green (2000) [45]; Bodet (2009) [46]
	Attractiveness of stadium color and decoration (PSQ2)	
	Overall cleanliness of the stadium (PSQ3)	
	Functionality diversity of stadium screens (PSQ4)	
	Stadium audio quality (PSQ5)	
	Number of restrooms in the stadium (PSQ6)	
	Comfort of stadium seating (PSQ7)	
	Quality of the playing field (PSQ8)	
	Clarity of spectators’ viewing lines (PSQ9)	
	Clarity of stadium signage (PSQ10)	
	Scientific management of spectator flow (PSQ11)	
	Variety of food and beverage options (PSQ12)	
	Cost-performance of food and beverages (PSQ13)	
	Variety of fan merchandise (PSQ14)	
	Cost-performance of fan merchandise (PSQ15)	
	Courtesy of stadium staff (e.g., ticket sellers, vendors, ushers) (PSQ16)	
	Adequacy of stadium staff numbers (PSQ17)	
	Speed at which stadium staff address spectators’ needs (PSQ18)	
**Spectator Satisfaction**	My decision to attend this match was the right one (S1)	Yoshida and James (2010) [22]； Sarstedt et al. (2014) [32]; Van Leeuwen et al. (2002) [47]
	The live match met my spectating needs (S2)	
	Overall, I am satisfied with the stadium services (S3)	
	The stadium services met my needs (S4)	
**Spectator Trust**	The club prioritizes improving match quality (T1)	Kim and Jeong (2025) [48] Wu et al. (2012) [49]
	The club considers fans’ interests when making major decisions (T2)	
	The club can be trusted to do the right thing (T3)	
**Team Identification**	The long-term success of the club I support is very important to me (TI1)	Tsigilis et al. (2023) [50]; Bodet and Bernache (2011) [51]; Theodorakis et al. (2010) [52]; Argan et al. (2019) [53]
	I feel a strong sense of belonging to the club I support (TI2)	
	When the team I support performs poorly, I often feel frustrated (TI3)	
	I wear or display symbols that represent the club’s identity when watching matches (TI4)	
	When others criticize the team, it feels like they are criticizing me personally (TI5)	
**Spectator Loyalty**	I will continue to support the club even when its performance is poor (L1)	Kim and Trail (2011) [26] Byon et al. (2013) [16]; Hennig-Thurau et al. (2002) [54]; Yoshida and James (2010) [22]
	I consistently follow the club’s developments (e.g., fixtures, transfers) (L2)	
	Attending the club’s matches next season is part of my future plans (L3)	
	I would recommend attending the club’s matches to others (L4)	
	I will continue to purchase the club’s merchandise or memorabilia (L5)	

### Survey

This study was conducted in accordance with the Declaration of Helsinki. Ethical approval was obtained from the Ethics Committee of Guangzhou Sport University (Approval No. 2025LCLL-143). Informed consent was obtained from all participants prior to data collection. Data were collected at stadium sites with the assistance of trained research personnel. Before administering the questionnaire, participants were informed of the study objectives and asked to confirm their willingness to participate. In addition, the questionnaire included an informed consent statement, and all respondents who completed the survey indicated that they had been fully informed and had consented to participate.

The formal survey recruitment period began on 14/06/2025 and ended on 02/08/2025. During this period, three CSL matches were selected for on-site survey administration, and members of the research team attended the stadiums to collect data from spectators. The matches included: Shenzhen Peng City FC (home) vs. Shanghai Shenhua FC (away) on June 14, 2025; Shenzhen Peng City FC (home) vs. Qingdao Hainiu FC (away) on June 19, 2025; and Meizhou Hakka FC (home) vs. Shanghai Port FC (away) on August 2, 2025. A total of 320 questionnaires were collected. After excluding 42 invalid responses (e.g., identical answers across all items or substantial missing data), 278 valid questionnaires were retained, yielding a valid response rate of 86.9%. Of the respondents, 256 were male and 22 were female. The age distribution was as follows: 87 respondents were under 20 years old, 80 were aged 21–30, 72 were aged 31–40, 30 were aged 41–50, 8 were aged 51–60, and 1 was over 60. The original data are in S1 Dataset.

### Data analysis

SPSS 22.0 was used to perform Cronbach’s alpha reliability tests, item analysis, and correlation analysis. Structural equation modeling (SEM) was used to assess model fit and estimate the path coefficients among latent variables. The PROCESS macro (Version 4.2) tested the mediating effects. AMOS 23.0 calculated model fit indices, including the chi-square to degrees of freedom ratio (χ²/df), root mean square error of approximation (RMSEA), goodness-of-fit index (GFI), adjusted goodness-of-fit index (AGFI), normed fit index (NFI), relative fit index (RFI), incremental fit index (IFI), Tucker–Lewis index (TLI), and comparative fit index (CFI).

## Results

### Reliability and validity of the measurement items

After collecting the formal survey data, the reliability and validity of the measurement scales were assessed. First, internal consistency was examined using Cronbach’s alpha, with values above 0.7 indicating acceptable reliability [55]. As shown in Table 3, the Cronbach’s alpha coefficients for core product quality, peripheral product quality, satisfaction, trust, team identification, and loyalty were 0.862 (17 items), 0.896 (18 items), 0.884 (4 items), 0.806 (3 items), 0.781 (5 items), and 0.841 (5 items), respectively. Second, the homogeneity and discrimination of the items were evaluated using item–total correlations and high–low group comparisons. As shown in Table 2, all item–total correlations exceeded the recommended threshold of 0.4. Furthermore, the high–low group comparisons revealed significant differences for all items (*p* < 0.01).

**Table 2:** The correlation coefficient and factor loading between items and respective constructs.

Items	R	SFL	Items	R	SFL	Items	R	SFL
CSQ1	0.553^***^	0.620	PSQ2	0.654^***^	0.814	S2	0.896^***^	0.904
CSQ2	0.497^***^	0.697	PSQ3	0.629^***^	0.836	S3	0.879^***^	0.809
CSQ3	0.534^***^	0.728	PSQ4	0.655^***^	0.767	S4	0.819^***^	0.701
CSQ4	0.626^***^	0.863	PSQ5	0.693^***^	0.805	T1	0.808^***^	0.673
CSQ5	0.607^***^	0.752	PSQ6	0.601^***^	0.671	T2	0.898^***^	0.954
CSQ6	0.565^***^	0.782	PSQ7	0.593^***^	0.517	T3	0.844^***^	0.683
CSQ7	0.633^***^	0.899	PSQ8	0.667^***^	0.738	TI1	0.743^***^	0.706
CSQ8	0.656^***^	0.887	PSQ9	0.675^***^	0.864	TI2	0.747^***^	0.701
CSQ9	0.647^***^	0.862	PSQ10	0.680^***^	0.846	TI3	0.680^***^	0.553
CSQ10	0.571^***^	0.568	PSQ11	0.649^***^	0.575	TI4	0.808^***^	0.751
CSQ11	0.440^***^	0.489	PSQ12	0.585^***^	0.735	TI5	0.695^***^	0.569
CSQ12	0.522^***^	0.559	PSQ13	0.433^***^	0.756	L1	0.793^***^	0.772
CSQ13	0.546^***^	0.562	PSQ14	0.554^***^	0.829	L2	0.846^***^	0.859
CSQ14	0.524^***^	0.615	PSQ15	0.558^***^	0.817	L3	0.792^***^	0.711
CSQ15	0.524^***^	0.860	PSQ16	0.600^***^	0.796	L4	0.793^***^	0.684
CSQ16	0.556^***^	0.905	PSQ17	0.597^***^	0.846	L5	0.680^***^	0.541
CSQ17	0.497^***^	0.585	PSQ18	0.572^***^	0.743	—	—	—
PSQ1	0.601^***^	0.730	S1	0.856^***^	0.825	—	—	—

Note: *** represents *P* < 0.01; the item codes are presented in Table 1.

**Table 3:** Testing the Reliability and Validity of the Measurement Model.

Latent Variable		Core product quality	Peripheral product quality	Satisfaction	Trust	Team identification	Loyalty
**Fit Indices**	**χ²/df**	1.842	8.003	26.463	3.866	6.467	10.222
	**RMSEA**	0.055	0.159	0.303	0.102	0.140	0.182
	**GFI**	0.994	0.973	0.909	0.991	0.955	0.927
	**AGFI**	0.968	0.866	0.545	0.944	0.865	0.780
	**NFI**	0.978	0.944	0.921	0.987	0.918	0.911
	**RFI**	0.935	0.832	0.763	0.962	0.836	0.823
	**IFI**	0.990	0.951	0.924	0.990	0.930	0.919
	**TLI**	0.969	0.850	0.770	0.971	0.858	0.837
	**CFI**	0.990	0.950	0.923	0.990	0.929	0.919
**Cronbach’s alpha**		0.862	0.896	0.884	0.806	0.781	0.841
**AVE**		0.537	0.586	0.661	0.610	0.437	0.520
**CR**		0.950	0.962	0.886	0.820	0.793	0.841

Third, confirmatory factor analysis (CFA) was conducted to evaluate the measurement model. Fit indices such as χ²/df, RMSEA, and AGFI are sensitive to sample sizes, whereas incremental fit indices—particularly IFI and CFI—are less affected by sample size, with CFI being especially robust [56]. As shown in Table 3, the IFI and CFI values for all measurement models exceeded the recommended threshold of 0.9. Fourth, convergent and discriminant validity were assessed using standardized factor loadings (SFL), average variance extracted (AVE), and composite reliability (CR), with recommended thresholds of >0.5, > 0.5, and >0.7, respectively [55,57]. As shown in Tables 2 and 3, most factor loadings exceeded 0.5, with only one item (CSQ11 = 0.489) falling slightly below the threshold. AVE values for all constructs exceeded 0.5 except team identification (AVE = 0.437), while all CR values exceeded 0.7. Prior research suggests that convergent validity remains acceptable when AVE is below 0.5 but CR exceeds 0.6 [58]. The AVE for team identification was marginally below the recommended level, but its CR value (0.793) exceeded 0.6. Fifth, Harman’s single-factor test was conducted to assess potential common method bias. The first unrotated factor had an initial eigenvalue of 11.810 and accounted for 22.711% of the total variance, which was well below the recommended threshold of 50%, indicating that common method bias was not a serious concern. These results indicate that the scales demonstrate satisfactory reliability and validity.

### Structural model and hypothesis testing

When a scale includes a large number of items, item parceling can be applied by combining multiple items into a single indicator using their mean or summed score [59]. Given the relatively large number of indicators for league core and peripheral product quality, this study employed parceling to simplify the model and enhance the clarity of structural analysis. Specifically, items for each construct were averaged and represented as a single observed variable. The same procedure was applied to spectator satisfaction, trust, team identification, and loyalty.

**Fig 2:**
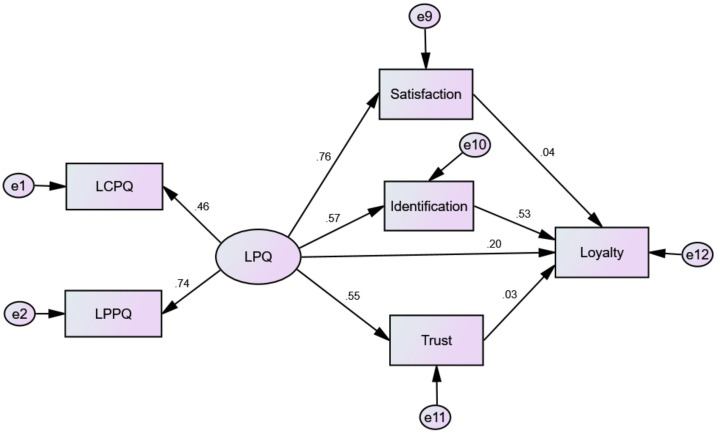
Structural model among the latent variables. Note: Abbreviations for variable names are shown in Table 1.

As shown in Table 4, the χ²/df, RMSEA, GFI, AGFI, NFI, RFI, IFI, TLI, and CFI values all met the recommended thresholds, indicating a good fit between the proposed theoretical model and the survey data. As shown in Table 5 and Fig 2, the standardized path coefficients for LPQ → Satisfaction, LPQ → Trust, LPQ → Identification, and Identification → Loyalty were all statistically significant (*p* < 0.01). These results indicate that league product quality has significant direct effects on spectator satisfaction, trust, and team identification, while team identification significantly predicts loyalty. Thus, Hypotheses H1, H2, H3, and H6 were supported. In contrast, the path coefficients for LPQ → Loyalty, Trust → Loyalty, and Satisfaction → Loyalty were not statistically significant (*p* > 0.05), indicating that league product quality, satisfaction, and trust do not exert direct effects on loyalty. Hypotheses H4, H5, and H7 were rejected.

**Table 4:** Structural model fit assessment.

Indicators		Recommended Criteria	Model value
**Absolute Fit Indices**	χ²/df	< 3.0	1.719
	RMSEA	< 0.1	0.051
	GFI	> 0.9	0.988
	AGFI		0.958
**Incremental Fit Indices**	NFI	> 0.9	0.979
	RFI		0.948
	IFI		0.991
	TLI		0.978
	CFI		0.991

**Table 5:** Path coefficient in the structural model.

Path	Standardized Estimates	S.E.	C.R.	P	Hypothesis
**H1: LPQ → Satisfaction**	0.759	0.282	6.725	0.000	Support
**H2: LPQ → Trust**	0.549	0.281	5.918	0.000	Support
**H3: LPQ → Identification**	0.570	0.264	6.030	0.000	Support
**H4: Satisfaction → Loyalty**	0.038	0.110	0.407	0.684	Reject
**H5: Trust → Loyalty**	0.029	0.057	0.486	0.627	Reject
**H6: Identification → Loyalty**	0.534	0.064	8.822	0.000	Support
**H7: LPQ → Loyalty**	0.205	0.425	1.423	0.155	Reject

Note: LPQ = League Product Quality.

Table 6 presents the mediating effects of spectator satisfaction, trust, and team identification. The indirect effects for the LPQ → Satisfaction → Loyalty and LPQ → Trust → Loyalty were 0.065 and 0.028, respectively. The 95% bootstrap confidence interval for each of the two pathways included zero, indicating that satisfaction and trust do not significantly mediate the relationship between league product quality and spectator loyalty. The indirect effect for LPQ → Team identification → Loyalty is 0.218, with its 95% Bootstrap confidence interval not including zero, indicating a significant mediating effect. Therefore, Hypotheses H8a and H8b are rejected, and H8c is supported.

**Table 6:** Test of mediating effects of satisfaction, trust and team identification.

	Effect size	BootSE	Boot LLCI	Boot ULCI	Hypothesis
**Total mediating effect**	0.311	0.042	0.227	0.393	—
**H8a: LPQ → Satisfaction → Loyalty**	0.065	0.033	−0.001	0.133	Reject
**H8b: LPQ → Trust → Loyalty**	0.028	0.028	−0.027	0.083	Reject
**H8c: LPQ → Team Identification → Loyalty**	0.218	0.042	0.140	0.302	Support

Note: LPQ = League Product Quality.

### Post-hoc exploratory SEM

**Fig 3:**
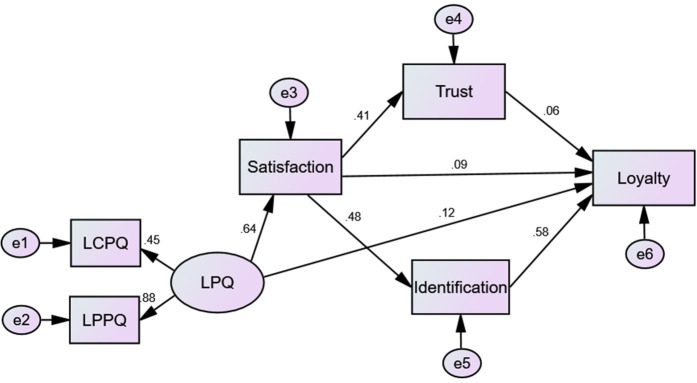
The structural paths of the sequential model.

The main model treated satisfaction, trust, and team identification as parallel mediators. However, prior studies have suggested that these variables may also operate sequentially. Based on the findings of Lee and Kang [60] and Huang and Kim [61], a post-hoc exploratory SEM was conducted to examine the sequential ordering of these variables within the LPQ-to-loyalty pathway (see Fig. 3). The model fit indices were as follows: χ²/df = 5.567, RMSEA = 0.128, GFI = 0.959, AGFI = 0.857, NFI = 0.933, RFI = 0.833, IFI = 0.945, TLI = 0.859, and CFI = 0.943. Overall, the sequential model showed a marginally acceptable fit level, although inferior to the main model.

As shown in Table 7, satisfaction exerted a significant positive effect on both trust and team identification (*p* < 0.05). Consistent with the main model, neither satisfaction nor trust significantly predicted loyalty (*p* > 0.05); team identification had a significant positive effect on loyalty (*p* < 0.05). Further, the chain mediation analysis revealed that the pathway LPQ → Satisfaction → Trust → Loyalty was not statistically significant (BootLLCI = −0.007, BootULCI = 0.052). In contrast, the pathway LPQ → Satisfaction → Team Identification → Loyalty was significant (BootLLCI = 0.061, BootULCI = 0.176), with an effect size of 0.112.

**Table 7:** Path coefficients in sequential model.

Path	Standardized Estimates	S.E.	C.R.	P
**LPQ → Satisfaction**	0.644	0.262	6.324	0.000
**Satisfaction → Trust**	0.407	0.067	7.420	0.000
**Satisfaction → Identification**	0.476	0.059	9.007	0.000
**Identification → Loyalty**	0.576	0.051	11.710	0.000
**Trust → Loyalty**	0.063	0.046	1.330	0.184
**Satisfaction → Loyalty**	0.089	0.083	1.253	0.210
**LPQ → Loyalty**	0.116	0.215	1.631	0.103

## Discussion

### Main findings

The first main finding of this study is that perceived league product quality has a significant direct effect on spectator satisfaction and trust, which is consistent with established research in sport marketing. For example, Greenwell et al. (2002) demonstrated that both core and peripheral product quality significantly influence spectator satisfaction in a minor ice hockey league [62]; Sarstedt et al. (2009) showed that peripheral service attributes—such as stadium facilities, cleanliness, catering services, and fan shops—play an important role in shaping spectators’ satisfaction [63]. Research by Schijns et al. (2016) on Dutch sports clubs confirms that perceived service quality has a significant effect on both satisfaction and trust [64]. These findings can be explained by the Expectation-Confirmation Theory and Social Exchange Theory. Satisfaction arises from the comparison between perceived performance and prior expectations. When league products perform at or above expected standards, spectators are more likely to form favorable affective reactions. In addition, stable and professional service delivery conveys signals of competence and reliability, thereby facilitating the development of trust between spectators and sport organizations.

The second main finding is that no significant direct effect of perceived league product quality on spectator loyalty. This finding contrasts with the prevailing view in sport marketing literature, which reports a significant direct relationship between quality and loyalty [16,65,66]. One plausible explanation lies in the fact that, unlike conventional consumer markets, where product switching is frequent and consumer preferences change rapidly, the sport exhibits a distinctive phenomenon: individuals develop deep and enduring attachments to teams, athletes, and even sporting traditions. Fan loyalty in sport is not merely a transactional preference for a brand or product; rather, it emerges from the complex interplay of psychological identification, emotional attachment, social belonging, and so on [67].

The third main finding of this study is that team identification is a significant predictor of spectator loyalty, this result is consistent with the prevailing view in the literature: fans with higher levels of identification are more likely to engage in loyalty-related behaviors, including repeat attendance, media consumption, and supportive actions [68,69]. Furthermore, team identification serves a key mediating mechanism in the process through which external stimuli (product attributes) translate into enduring behavioral tendencies (loyalty). This process can be explained through Social Identity Theory. According to this theory, individuals derive part of their self-concept from their membership in social groups and tend to categorize themselves and others into “in-groups” and “out-groups”. Professional sports competitions are jointly produced by two opposing teams, providing an ideal objective for spectators to categorize themselves into a group that highlights their own characteristics and satisfies their self-concept or self-image. Ongoing competition reinforces an “us versus them” mindset, strengthening in-group favoritism and shaping fans’ perceptions of rival teams and their supporters [70].

Peripheral services, such as stadium facilities, match-day atmosphere, and fan services, also play an important role in strengthening spectators’ team identification [71,72]. Within the stadium environment, spectators, players, and staff collectively create a temporary community that fosters both emotional and psychological bonds between fans and the team, gradually cultivating a “home-away-from-home” feeling and enhancing identification with the club [73]. Match-day atmosphere includes stadium decorations with club colors, wearing team colors, Tifos, and the collective singing of team anthems. These activities enable individuals to experience a heightened sense of energy and unity, thereby fostering a sense of belonging and communal identity [74]. Furthermore, tangible goods that spectators can take away—such as souvenirs or mailed memorabilia—serve as continuous reminders of the experience, further reinforcing their identification with the team [75].

The fourth main finding of this study is that the sequential path Product Quality → Satisfaction → Team Identification → Loyalty is statistically significant. This finding aligns with Zhao et al. (2024), who reported that team quality significantly influences both satisfaction and fan attachment, with satisfaction serving as a positive mediator [76]. The present findings are also consistent with the Psychological Continuum Model proposed by Funk and James (2001), which posits that committed fandom develops through a progressive psychological process from awareness to attraction, then to emotional attachment, and ultimately to loyalty [77]. The CSL was established in 2004, and only 33% of CSL fans in the 2019 season had followed the league for more than five years [78]. Therefore, many spectators may still be in the early stages of psychological connection with the league. In this context, loyalty may depend more heavily on positive consumption experience, and repeated satisfactory experiences derived from high-quality league products may strengthen team identification and subsequently foster loyal behaviors.

### Limitations

Several limitations should be acknowledged. First, the study has a notable gender imbalance. Female spectators accounted for about 8% (n = 22) of the sample. Won et al. (2025), in a study of Japan’s B League and J League, identified gender differences in spectators’ motivations, including team identification, game attractiveness, players, and facilities [79]. However, the small number of female participants made robust multi-group SEM analysis difficult, as model estimation may become unstable when group sample sizes fall below 50 [80]. Therefore, this study’s findings are primarily generalizable to male spectators. Future research should recruit more female participants to assess potential gender differences in the relationships among variables.

Second, the study’s proposed model was tested using data from only three CSL matches, with surveyed spectators primarily drawn from Guangdong Province. Consequently, the findings may not generalize to spectators from other regions or across different competitive contexts. Future research should recruit samples from a broader range of CSL venues and across multiple seasons to further examine the stability of the proposed model.

Third, several procedural controls were implemented to mitigate common method bias, including expert review and a pilot survey to improve item validity, clear questionnaire instructions, and respondent anonymity. Nevertheless, the study relied on self-reported, cross-sectional data collected at a single point in time. These controls cannot fully eliminate common method bias. Future research should employ multi-source data (e.g., objective behavioral measures) and longitudinal designs to enhance the robustness of the findings.

Fourth, this study provided preliminary evidence for the sequential pathway LPQ → Satisfaction → Team Identification → Loyalty. However, this pattern should not be regarded as universal. McDonald et al. (2016) suggested that, for fans with a family tradition of supporting a particular team or those influenced by existing fan communities, team identification may emerge without first passing through a stage of satisfaction with league product quality [81]. Future research should more rigorously examine the sequential relationships among variables, particularly across different fan segments (e.g., peripheral, marginal, and core fans).

## Conclusion

The empirical findings indicate that perceived league product quality significantly enhances spectator satisfaction, trust, and team identification, but does not directly affect spectator loyalty. Team identification has a direct and significant effect on loyalty, serving as a mediator between league product quality and loyalty. The theoretical contribution of this study lies in clarifying the structural pathways within the “cognition–affect–behavior” framework of spectator attitudes, thereby extending understanding of loyalty formation mechanisms in professional football in China. Additionally, the study provides clear practical implications for the CSL. League operations and product design should not focus solely on enhancing the attractiveness of the match and service experience, but should also prioritize cultivating team identification as a key strategy.

## Supporting information

S1 Dataset The original Data used for this study. (XLSX)
