# Supplementary material for: The relationships among perceived league product quality, spectator satisfaction, trust, team identification, and loyalty in the Chinese Super League
Source: PLoS One. 2026 Jun 8;21(6):e0350647. doi: 10.1371/journal.pone.0350647 (PMC13245865; doi:10.1371/journal.pone.0350647)
Supplement: Attachment — Submitted filename: Point by point response to reviewer.docx [file pone.0350647.s003.docx]

**Dear editor and reviewer:**

We sincerely thank you for your time and effort in reviewing our manuscript. In response to your valuable comments, we have made substantial revisions, which are highlighted in red in the revised manuscript. We have prepared a detailed point-by-point responses to address the comments of reviewer 1 and reviewer 2.

**Reviewer 1 comments**

**Comment 1**

The conceptual alignment of the model needs clarification. The manuscript focuses on perceived league product quality, but several downstream constructs are framed at the club level. In particular, the trust items refer to the club, and both team identification and loyalty are also club-oriented. The paper therefore needs a clearer explanation of why a league-level perception is expected to predict club-level trust, identification, and loyalty.

**Response**

Thank you for this insightful comment. We have clarified the conceptual alignment of the model. This can be found in lines 120–126 of the revised manuscript, as follows:

*In this study, although league product quality is conceptualized at the league level, professional leagues inherently operate as nested systems in which clubs function as constituent units that both compete and collaborate. The league product, encompassing both core and peripheral attributes, is primarily delivered through clubs. Therefore, spectators’ perceptions of league product quality are largely formed from their evaluations of these club-delivered elements. It is theoretically reasonable to expect that perceived league product quality influences club-level psychological and behavioral outcomes*.

**Comment 2**

The reporting of the measurement model is not sufficient for a structural equation study. The manuscript provides Cronbach’s alpha, item-total correlations, and high-low group comparisons, but this does not adequately establish construct validity. The authors need to report the measurement model more fully and explain more clearly how league product quality was represented in the SEM.

**Response**

1. We have added the CFA results, the standardized factor loadings, average variance extracted (AVE), and composite reliability (CR) of the measurement model, further demonstrating that the measurement model has good construct validity. See Tables 2 and 3, as well as lines 259–271, in the revised manuscript.
2. We have more clearly explained how league product quality is represented in the structural equation model. See lines 280–285 of the revised manuscript.

**Comment 3**

There are internal inconsistencies in the statistical reporting. The abstract and Methods section report different software versions. In addition, the Data analysis subsection ends with an incomplete sentence (“In addition.”).

Table 2 should be checked very carefully. The uploaded dataset uses regular variable names such as L1–L5 and TI1–TI5, whereas Table 2 contains labels such as CL1, CL2, CL5, CL6, CL7, and L7. This looks like a reporting or editing problem and should be corrected throughout the manuscript.
One reliability coefficient also appears to require verification. The uploaded dataset is generally consistent with the sample description in the manuscript, which is a strength. However, the reported Cronbach’s alpha for core product quality should be recalculated and checked against the uploaded data.

**Response**

Thank you for pointing out these editorial errors.

1. We have modified the software version reported in the abstract. This can be found in line 49 of the revised manuscript.
2. In the methods section, we removed the redundant “In addition”.
3. We proofread the variable names in Table 2 to ensure consistency with the uploaded dataset.
4. We recalculated Cronbach’s alpha coefficient for core product quality. This can be found in line 253 of the revised manuscript.

**Comment 4**

The limits of the sample need to be acknowledged more explicitly. The formal sample was drawn from only three matches, and the gender distribution is highly imbalanced. The manuscript should therefore avoid broad claims about all CSL spectators.

**Response**

In the limitations section, we have clearly acknowledged the limitations arising from the unbalanced gender distribution and the small number of matches surveyed. These revisions can be found in lines 366–375 of the revised manuscript

**Comment 5**

The language is understandable, but the manuscript still requires editing. There are several grammatical and editorial problems that should be corrected before further consideration.

**Response**

We have carefully reviewed the whole manuscript and corrected the grammatical and editorial issues.

**Reviewer 2 comments**

**Comment 1**

There are a few areas where the manuscript could be further strengthened. The theoretical contribution could be articulated more explicitly, particularly in how it positions the findings in relation to existing research. From a methodological perspective, including additional details on the measurement model (e.g., confirmatory factor analysis results, factor loadings, and indicators of convergent and discriminant validity, such as AVE and CR) would further enhance the study's robustness. It may also be helpful to briefly address potential common method bias given the use of self-reported, cross-sectional data.

**Response**

1. We have more clearly articulated the theoretical contributions of this study in Discussion and Conclusion section. See lines 344-346, and 391–394 of the revised manuscript.
2. In the results section, we have added the CFA results, the standardized factor loadings, average variance extracted (AVE), and composite reliability (CR) of the measurement model, further demonstrating that the measurement model has good construct validity. See Tables 2 and 3, as well as lines 259–271, in the revised manuscript.

3. Regarding the common method bias, we conducted Harman's single-factor test. The results showed that the variance explained by the first unrotated factor was 22.7%, which did not exceed the 50% threshold. In addition, we discussed this issue in the limitations section. See lines 272–274 and 376-382 of the revised manuscript.

**Comment 2**

Additionally, expanding the discussion of limitations—especially regarding sample characteristics and generalizability—would improve transparency. The discussion section could also benefit from slightly deeper engagement with recent literature on team identification and sport consumer behaviour. For instance, recent research (https://doi.org/10.1371/journal.pone.0343795) highlights the mediating role of psychological constructs such as fan loyalty, which may help contextualize the present findings.

**Response**

1. In the Limitations section, we have clearly acknowledged the limitations arising from the unbalanced gender distribution and the small number of matches surveyed. These revisions can be found in lines 366–375 of the revised manuscript.
2. In the Discussion section, we added the latest research literature on team identification and sports consumption behavior, such as Akgül et al. (2026) and Kwon et al. (2022). See lines 336–337, 340-343, and 360-361 in the revised manuscript.
