# Supplementary material for: The relationships among perceived league product quality, spectator satisfaction, trust, team identification, and loyalty in the Chinese Super League
Source: PLoS One. 2026 Jun 8;21(6):e0350647. doi: 10.1371/journal.pone.0350647 (PMC13245865; doi:10.1371/journal.pone.0350647)
Supplement: Attachment — Submitted filename: Point_by_point_response_to_reviewer_auresp_2.docx [file pone.0350647.s004.docx]

**Dear editor and reviewer:**

We sincerely thank you for your time and effort in reviewing our manuscript. In response to your valuable comments, we have made substantial revisions, which are highlighted in red in the revised manuscript. We have prepared a detailed point-by-point responses.

**Comment 1**

Please include the full value for the variance explained by the first unrotated factor in Harman's test.

**Response**

We apologize for the omission of this specific value in the previous version. In the revised manuscript, we have now reported the full variance explained by the first unrotated factor in Harman’s single-factor test. See Lines 271–274 of the revised manuscript.

The specific modifications are as follows:

*Fifth, Harman’s single-factor test was conducted to assess potential common method bias. The first unrotated factor had an initial eigenvalue of 11.810 and accounted for 22.711% of the total variance, which was well below the recommended threshold of 50%, indicating that common method bias was not a serious concern.*

**Comment 2**

Structural Relationships: Post-hoc structural equation modelling could be used to determine if satisfaction, trust and team identification are in a sequential relationship.

**Response**

Thank you for this valuable suggestion. We conducted a post-hoc structural equation modeling analysis to examine the potential sequential relationships among satisfaction, trust, and team identification. The corresponding results and discussion have been added to the revised manuscript. Please see Lines 310–326, 376–387, and 408–414 of the revised manuscript. In addition, the Abstract has been revised accordingly (see Lines 59–60).

**Comment 3**

Gender Imbalance: With the 8% female response rate, should you consider a gender split or discussion in the Discussion section?

**Response**

We thank the Editor and Reviewer for raising this important issue. After careful consideration, we did not conduct a multi-group SEM analysis because the female subsample falls below the recommended threshold for such analyses. Previous research suggests that model estimation may become unstable when group sample sizes are below 50 (Somer et al., 2024).

We have explicitly acknowledged this limitation in the revised manuscript. Specifically, we note that the findings are primarily generalizable to male spectators and recommend that future research recruit larger female samples to formally examine potential gender differences. Please see Lines 389–396 of the revised manuscript.

**Comment 4**

Citation Error: Please check the date for Akgül et al. (2026).

**Response**

We have deleted this reference.

**Comment 5**

Generalisability: Please add a recommendation to future research to test the model with various CSL venues and seasons.

**Response**

We have added this content in the revised manuscript. See lines 399-401 of the revised manuscript.
