# Supplementary material for: The relationships among perceived league product quality, spectator satisfaction, trust, team identification, and loyalty in the Chinese Super League
Source: PLoS One. 2026 Jun 8;21(6):e0350647. doi: 10.1371/journal.pone.0350647 (PMC13245865; doi:10.1371/journal.pone.0350647)
Supplement: Peer review history

# Peer review: Decision Letter 0

**DOI:** 10.1371/journal.pone.0350647.r001

**Authors:** Clementswami Sukumaran

26 Mar 2026

PONE-D-26-07142

The Relationships Among Perceived League Product Quality, Spectator Satisfaction, Trust, Team Identification, and Loyalty in the Chinese Super League

PLOS One

Dear Dr. Tian,

Thank you for submitting your manuscript to PLOS ONE. After careful consideration, we feel that it has merit but does not fully meet PLOS ONE’s publication criteria as it currently stands. Therefore, we invite you to submit a revised version of the manuscript that addresses the points raised during the review process.

Please submit your revised manuscript by May 10 2026 11:59PM. If you will need more time than this to complete your revisions, please reply to this message or contact the journal office at plosone@plos.org. When you're ready to submit your revision, log on to https://www.editorialmanager.com/pone/ and select the 'Submissions Needing Revision' folder to locate your manuscript file.

Please include the following items when submitting your revised manuscript:

- A letter that responds to each point raised by the academic editor and reviewer(s). You should upload this letter as a separate file labeled 'Response to Reviewers'.
- A marked-up copy of your manuscript that highlights changes made to the original version. You should upload this as a separate file labeled 'Revised Manuscript with Track Changes'.
- An unmarked version of your revised paper without tracked changes. You should upload this as a separate file labeled 'Manuscript'.

If you would like to make changes to your financial disclosure, please include your updated statement in your cover letter. Guidelines for resubmitting your figure files are available below the reviewer comments at the end of this letter.

If applicable, we recommend that you deposit your laboratory protocols in protocols.io to enhance the reproducibility of your results. Protocols.io assigns your protocol its own identifier (DOI) so that it can be cited independently in the future. For instructions see: https://journals.plos.org/plosone/s/submission-guidelines#loc-laboratory-protocols. Additionally, PLOS ONE offers an option for publishing peer-reviewed Lab Protocol articles, which describe protocols hosted on protocols.io. Read more information on sharing protocols at https://plos.org/protocols?utm_medium=editorial-email&utm_source=authorletters&utm_campaign=protocols.

As the corresponding author, your ORCID iD is verified in the submission system and will appear in the published article. PLOS supports the use of ORCID, and we encourage all coauthors to register for an ORCID iD and use it as well. Please encourage your coauthors to verify their ORCID iD within the submission system before final acceptance, as unverified ORCID iDs will not appear in the published article. *Only* the individual author can complete the verification step; PLOS staff *cannot* verify ORCID iDs on behalf of authors.

We look forward to receiving your revised manuscript.

Kind regards,

Clementswami Sukumaran, PhD

Academic Editor

PLOS One

Journal Requirements:

When submitting your revision, we need you to address these additional requirements.

1. Please ensure that your manuscript meets PLOS ONE's style requirements, including those for file naming. The PLOS ONE style templates can be found at

https://journals.plos.org/plosone/s/file?id=wjVg/PLOSOne_formatting_sample_main_body.pdf and

https://journals.plos.org/plosone/s/file?id=ba62/PLOSOne_formatting_sample_title_authors_affiliations.pdf

2. Please include captions for your Supporting Information files at the end of your manuscript, and update any in-text citations to match accordingly. Please see our Supporting Information guidelines for more information: http://journals.plos.org/plosone/s/supporting-information.

3. If the reviewer comments include a recommendation to cite specific previously published works, please review and evaluate these publications to determine whether they are relevant and should be cited. There is no requirement to cite these works unless the editor has indicated otherwise.

**Additional Editor Comments:**

-->Reviewer 1

Explain the theoretical contribution and location of findings in existing literature.

Present the full measurement model (CFA, factor loadings, CR, AVE) to determine convergent and discriminant validity.

Combat possible common method bias since the data is cross-sectional and self-reported.

Consider the recent literature (e.g., https://doi.org/10.1371/<wbr/>journal.pone.0343795) and extend the argument on limitations and especially on sample generalizability.

Reviewer 2

Make clear conceptual correspondence: clarify the rationale behind the predictive value of conceptual congruency between league perceived quality and club-level trust, identification, and loyalty.

Offer a full-fledged measurement model (CFA, factor loadings, CR, AVE) and explain how the quality of league products was modeled in the SEM.

Right internal inconsistencies, version of software in abstract, unfinished sentence in data analysis section.

Match labels in Table 2 with the names of variables in the dataset.

Check the reported Cronbachs alpha of core product quality against data.

Admit to sample limitations (three matches, unequal gender) and do not make generalizations about all CSL spectators.

Correct grammar and typing mistakes.

[Note: HTML markup is below. Please do not edit.]

Reviewers' comments:

Reviewer's Responses to Questions

**Comments to the Author**

1. Is the manuscript technically sound, and do the data support the conclusions?

The manuscript must describe a technically sound piece of scientific research with data that supports the conclusions. Experiments must have been conducted rigorously, with appropriate controls, replication, and sample sizes. The conclusions must be drawn appropriately based on the data presented.

Reviewer #1: Partly

Reviewer #2: Partly

**********

2. Has the statistical analysis been performed appropriately and rigorously?

Reviewer #1: No

Reviewer #2: Yes

**********

3. Have the authors made all data underlying the findings in their manuscript fully available?

The PLOS Data policy requires authors to make all data underlying the findings described in their manuscript fully available without restriction, with rare exception (please refer to the Data Availability Statement in the manuscript PDF file). The data should be provided as part of the manuscript or its supporting information, or deposited to a public repository. For example, in addition to summary statistics, the data points behind means, medians and variance measures should be available. If there are restrictions on publicly sharing data—e.g. participant privacy or use of data from a third party—those must be specified.

Reviewer #1: Yes

Reviewer #2: Yes

**********

4. Is the manuscript presented in an intelligible fashion and written in standard English?

PLOS ONE does not copyedit accepted manuscripts, so the language in submitted articles must be clear, correct, and unambiguous. Any typographical or grammatical errors should be corrected at revision, so please note any specific errors here.

Reviewer #1: No

Reviewer #2: No

**********

5. Review Comments to the Author

Please use the space provided to explain your answers to the questions above. You may also include additional comments for the author, including concerns about dual publication, research ethics, or publication ethics. (Please upload your review as an attachment if it exceeds 20,000 characters)

Reviewer #1: Thank you for the opportunity to review this manuscript. The topic is relevant, and the availability of an underlying dataset is a positive aspect. However, the manuscript still requires major revision before its technical soundness can be properly evaluated.

My main concerns are listed below.

The conceptual alignment of the model needs clarification. The manuscript focuses on perceived league product quality, but several downstream constructs are framed at the club level. In particular, the trust items refer to the club, and both team identification and loyalty are also club-oriented. The paper therefore needs a clearer explanation of why a league-level perception is expected to predict club-level trust, identification, and loyalty.

The reporting of the measurement model is not sufficient for a structural equation study. The manuscript provides Cronbach’s alpha, item-total correlations, and high-low group comparisons, but this does not adequately establish construct validity. The authors need to report the measurement model more fully and explain more clearly how league product quality was represented in the SEM.

There are internal inconsistencies in the statistical reporting. The abstract and Methods section report different software versions. In addition, the Data analysis subsection ends with an incomplete sentence (“In addition.”). These problems need to be corrected carefully because they reduce confidence in the rigor of the methods section.

Table 2 should be checked very carefully. The uploaded dataset uses regular variable names such as L1–L5 and TI1–TI5, whereas Table 2 contains labels such as CL1, CL2, CL5, CL6, CL7, and L7. This looks like a reporting or editing problem and should be corrected throughout the manuscript.

One reliability coefficient also appears to require verification. The uploaded dataset is generally consistent with the sample description in the manuscript, which is a strength. However, the reported Cronbach’s alpha for core product quality should be recalculated and checked against the uploaded data.

The limits of the sample need to be acknowledged more explicitly. The formal sample was drawn from only three matches, and the gender distribution is highly imbalanced. The manuscript should therefore avoid broad claims about all CSL spectators.

The language is understandable, but the manuscript still requires editing. There are several grammatical and editorial problems that should be corrected before further consideration.

Overall, this study has a potentially usable dataset and addresses a relevant topic, but the current version still has important problems in conceptual alignment, measurement reporting, and manuscript preparation. Major revision is therefore more appropriate than rejection at this stage.

Reviewer #2: This manuscript addresses a relevant and timely topic by examining the relationships among perceived league product quality, spectator satisfaction, trust, team identification, and loyalty within the ABC attitude framework. The integration of these constructs into a unified model is a clear strength, and the use of in-stadium spectator data provides valuable practical relevance. Overall, the study offers useful insights into sports consumer behaviour, particularly within the context of the Chinese Super League.

The manuscript is generally well-structured, and the analytical approach is appropriate. The findings—especially the central role of team identification—are interesting and have the potential to contribute meaningfully to the sport marketing literature.

There are, however, a few areas where the manuscript could be further strengthened. The theoretical contribution could be articulated more explicitly, particularly in how it positions the findings in relation to existing research. From a methodological perspective, including additional details on the measurement model (e.g., confirmatory factor analysis results, factor loadings, and indicators of convergent and discriminant validity, such as AVE and CR) would further enhance the study's robustness. It may also be helpful to briefly address potential common method bias given the use of self-reported, cross-sectional data.

Additionally, expanding the discussion of limitations—especially regarding sample characteristics and generalizability—would improve transparency. The discussion section could also benefit from slightly deeper engagement with recent literature on team identification and sport consumer behaviour. For instance, recent research (https://doi.org/10.1371/journal.pone.0343795) highlights the mediating role of psychological constructs such as fan loyalty, which may help contextualize the present findings.

Overall, this is a well-executed study with clear potential for publication. Addressing the points above would further strengthen the manuscript and enhance its clarity, methodological rigor, and theoretical positioning.

**********

6. PLOS authors have the option to publish the peer review history of their article (what does this mean?). If published, this will include your full peer review and any attached files.

If you choose “no”, your identity will remain anonymous but your review may still be made public.

**Do you want your identity to be public for this peer review?** For information about this choice, including consent withdrawal, please see our Privacy Policy.

Reviewer #1: No

Reviewer #2: No

**********

[NOTE: If reviewer comments were submitted as an attachment file, they will be attached to this email and accessible via the submission site. Please log into your account, locate the manuscript record, and check for the action link "View Attachments". If this link does not appear, there are no attachment files.]

To ensure your figures meet our technical requirements, please review our figure guidelines: https://journals.plos.org/plosone/s/figures

You may also use PLOS’s free figure tool, NAAS, to help you prepare publication quality figures: https://journals.plos.org/plosone/s/figures#loc-tools-for-figure-preparation.

NAAS will assess whether your figures meet our technical requirements by comparing each figure against our figure specifications.

# Author comment: Author response to Decision Letter 1

**DOI:** 10.1371/journal.pone.0350647.r002

11 Apr 2026

We have made substantial revisions to the manuscript in response to the reviewers' comments and have provided a point-by-point response.

Attachment Submitted filename: Point by point response to reviewer.docx

# Peer review: Decision Letter 1

**DOI:** 10.1371/journal.pone.0350647.r003

**Authors:** Clementswami Sukumaran

1 May 2026

PONE-D-26-07142R1

The Relationships among perceived league product quality, spectator satisfaction, trust, team identification, and loyalty in the Chinese Super League

PLOS One

Dear Dr. Tian,

Thank you for submitting your manuscript to PLOS ONE. After careful consideration, we feel that it has merit but does not fully meet PLOS ONE’s publication criteria as it currently stands. Therefore, we invite you to submit a revised version of the manuscript that addresses the points raised during the review process.

==============================

**ACADEMIC EDITOR:**

Dear Authors,

Thank you for your revisions. There are a couple of minor items to be addressed:

Common Method Bias: Please include the full value for the variance explained by the first unrotated factor in Harman's test.

Structural Relationships: Post-hoc structural equation modelling could be used to determine if satisfaction, trust and team identification are in a sequential relationship.

Gender Imbalance: With the 8% female response rate, should you consider a gender split or discussion in the Discussion section?

Citation Error: Please check the date for Akgül et al. (2026).

Generalisability: Please add a recommendation to future research to test the model with various CSL venues and seasons.

With these minor changes, the manuscript will be accepted.

==============================

Please submit your revised manuscript by Jun 15 2026 11:59PM. If you will need more time than this to complete your revisions, please reply to this message or contact the journal office at plosone@plos.org. When you're ready to submit your revision, log on to https://www.editorialmanager.com/pone/ and select the 'Submissions Needing Revision' folder to locate your manuscript file.

Please include the following items when submitting your revised manuscript:

- A letter that responds to each point raised by the academic editor and reviewer(s). You should upload this letter as a separate file labeled 'Response to Reviewers'.
- A marked-up copy of your manuscript that highlights changes made to the original version. You should upload this as a separate file labeled 'Revised Manuscript with Track Changes'.
- An unmarked version of your revised paper without tracked changes. You should upload this as a separate file labeled 'Manuscript'.

If you would like to make changes to your financial disclosure, please include your updated statement in your cover letter. Guidelines for resubmitting your figure files are available below the reviewer comments at the end of this letter.

If applicable, we recommend that you deposit your laboratory protocols in protocols.io to enhance the reproducibility of your results. Protocols.io assigns your protocol its own identifier (DOI) so that it can be cited independently in the future. For instructions see: https://journals.plos.org/plosone/s/submission-guidelines#loc-laboratory-protocols. Additionally, PLOS ONE offers an option for publishing peer-reviewed Lab Protocol articles, which describe protocols hosted on protocols.io. Read more information on sharing protocols at https://plos.org/protocols?utm_medium=editorial-email&utm_source=authorletters&utm_campaign=protocols.

As the corresponding author, your ORCID iD is verified in the submission system and will appear in the published article. PLOS supports the use of ORCID, and we encourage all coauthors to register for an ORCID iD and use it as well. Please encourage your coauthors to verify their ORCID iD within the submission system before final acceptance, as unverified ORCID iDs will not appear in the published article. *Only* the individual author can complete the verification step; PLOS staff *cannot* verify ORCID iDs on behalf of authors.

We look forward to receiving your revised manuscript.

Kind regards,

Clementswami Sukumaran, PhD

Academic Editor

PLOS One

Journal Requirements:

1. If the reviewer comments include a recommendation to cite specific previously published works, please review and evaluate these publications to determine whether they are relevant and should be cited. There is no requirement to cite these works unless the editor has indicated otherwise.

2. Please review your reference list to ensure that it is complete and correct. If you have cited papers that have been retracted, please include the rationale for doing so in the manuscript text, or remove these references and replace them with relevant current references. Any changes to the reference list should be mentioned in the rebuttal letter that accompanies your revised manuscript. If you need to cite a retracted article, indicate the article’s retracted status in the References list and also include a citation and full reference for the retraction notice.

[Note: HTML markup is below. Please do not edit.]

Reviewers' comments:

Reviewer's Responses to Questions

**Comments to the Author**

1. If the authors have adequately addressed your comments raised in a previous round of review and you feel that this manuscript is now acceptable for publication, you may indicate that here to bypass the “Comments to the Author” section, enter your conflict of interest statement in the “Confidential to Editor” section, and submit your "Accept" recommendation.

Reviewer #2: All comments have been addressed

Reviewer #3: All comments have been addressed

**********

2. Is the manuscript technically sound, and do the data support the conclusions?

The manuscript must describe a technically sound piece of scientific research with data that supports the conclusions. Experiments must have been conducted rigorously, with appropriate controls, replication, and sample sizes. The conclusions must be drawn appropriately based on the data presented.

Reviewer #2: Yes

Reviewer #3: Yes

**********

3. Has the statistical analysis been performed appropriately and rigorously?

Reviewer #2: Yes

Reviewer #3: Yes

**********

4. Have the authors made all data underlying the findings in their manuscript fully available?

The PLOS Data policy requires authors to make all data underlying the findings described in their manuscript fully available without restriction, with rare exception (please refer to the Data Availability Statement in the manuscript PDF file). The data should be provided as part of the manuscript or its supporting information, or deposited to a public repository. For example, in addition to summary statistics, the data points behind means, medians and variance measures should be available. If there are restrictions on publicly sharing data—e.g. participant privacy or use of data from a third party—those must be specified.

Reviewer #2: Yes

Reviewer #3: Yes

**********

5. Is the manuscript presented in an intelligible fashion and written in standard English?

PLOS ONE does not copyedit accepted manuscripts, so the language in submitted articles must be clear, correct, and unambiguous. Any typographical or grammatical errors should be corrected at revision, so please note any specific errors here.

Reviewer #2: Yes

Reviewer #3: Yes

**********

6. Review Comments to the Author

Please use the space provided to explain your answers to the questions above. You may also include additional comments for the author, including concerns about dual publication, research ethics, or publication ethics. (Please upload your review as an attachment if it exceeds 20,000 characters)

Reviewer #2: Dear author(s), thanks for your answers. All of my comments have been addressed. I do not have any further comments

Reviewer #3: Dear Authors,

I appreciate you taking the time to make improvements to the manuscript. Although many of the concerns have been resolved, there are some minor issues that need to be addressed.

Here are the comments and questions to be considered:

1. Statistical value omitted in Analysis of Common Method Bias

Concern: In the response to the common method bias, the variance explained by the first unrotated factor is presented as "\cdot" rather than a full percentage. This seems to be a typographical error or omission.

Query:

"Please report the variance explained by the first unrotated factor in Harman's single factor test. Right now, just seeing a "place holder".

2. Ambiguities in the Structure of Key Variables

Issue: The authors have mentioned the limitations of the measurement model, but the paper does not discuss the relationships between satisfaction, trust and team identification. These are presented as parallel constructs, but other research indicates they could be sequential.

Query:

"Have you explored whether post-hoc exploratory structural equation modeling (SEM) to see if satisfaction and trust operate sequentially? This would add to the theoretical insights into loyalty development processes."

3. Gender Disparity and No Solution

The manuscript mentions that 8% of the sample are women, however, no strategy (e.g., gender-based subanalysis) or mention of gender differences in future research is offered.

Query:

"In addition to noting the 8% female sample, would a gender-based sub-analysis or discussion of gender-based differences in the Discussion add to the manuscript?"

4. Unusual Year of Publication

Akgül et al. (2026) is mentioned in the Discussion, which is a future date. Please verify this reference.

Query:

"Please check the date for Akgül et al. The date (2026) looks like a future date. Please check the accuracy of the citation."

5. Generalizability Limited, Not Fully Addressed

Concerns: The sample size (only three matches in Guangdong Province) is discussed, but no recommendations for multi-site or longitudinal studies are explicitly stated.

Query:

"Could you include a line in the Conclusion section suggesting replication of the model across several CSL venues and seasons to increase generalisability of the results?"

Note; the incomplete reporting of the common method bias statistic is a notable flaw that should be resolved before final acceptance.

**********

7. PLOS authors have the option to publish the peer review history of their article (what does this mean?). If published, this will include your full peer review and any attached files.

If you choose “no”, your identity will remain anonymous but your review may still be made public.

**Do you want your identity to be public for this peer review?** For information about this choice, including consent withdrawal, please see our Privacy Policy.

Reviewer #2: No

Reviewer #3: No

**********

[NOTE: If reviewer comments were submitted as an attachment file, they will be attached to this email and accessible via the submission site. Please log into your account, locate the manuscript record, and check for the action link "View Attachments". If this link does not appear, there are no attachment files.]

To ensure your figures meet our technical requirements, please review our figure guidelines: https://journals.plos.org/plosone/s/figures

You may also use PLOS’s free figure tool, NAAS, to help you prepare publication quality figures: https://journals.plos.org/plosone/s/figures#loc-tools-for-figure-preparation.

NAAS will assess whether your figures meet our technical requirements by comparing each figure against our figure specifications.

# Author comment: Author response to Decision Letter 2

**DOI:** 10.1371/journal.pone.0350647.r004

10 May 2026

We revised the manuscript according to the editors' and reviewers' comments. Please see the "Point by point response to reviewer" document for our responses.

Attachment Submitted filename: Point_by_point_response_to_reviewer_auresp_2.docx

# Peer review: Decision Letter 2

**DOI:** 10.1371/journal.pone.0350647.r005

**Authors:** Clementswami Sukumaran

17 May 2026

The Relationships among perceived league product quality, spectator satisfaction, trust, team identification, and loyalty in the Chinese Super League

PONE-D-26-07142R2

Dear Dr. Tian,

We’re pleased to inform you that your manuscript has been judged scientifically suitable for publication and will be formally accepted for publication once it meets all outstanding technical requirements.

Within one week, you’ll receive an e-mail detailing the required amendments. When these have been addressed, you’ll receive a formal acceptance letter and your manuscript will be scheduled for publication.

An invoice will be generated when your article is formally accepted. Please note, if your institution has a publishing partnership with PLOS and your article meets the relevant criteria, all or part of your publication costs will be covered. Please make sure your user information is up-to-date by logging into Editorial Manager at Editorial Manager® and clicking the ‘Update My Information' link at the top of the page. For questions related to billing, please contact billing support.

If your institution or institutions have a press office, please notify them about your upcoming paper to help maximize its impact. If they’ll be preparing press materials, please inform our press team as soon as possible -- no later than 48 hours after receiving the formal acceptance. Your manuscript will remain under strict press embargo until 2 pm Eastern Time on the date of publication. For more information, please contact onepress@plos.org.

Kind regards,

Clementswami Sukumaran, PhD

Academic Editor

PLOS One

Additional Editor Comments (optional):

Reviewers' comments:

Reviewer's Responses to Questions

**Comments to the Author**

1. If the authors have adequately addressed your comments raised in a previous round of review and you feel that this manuscript is now acceptable for publication, you may indicate that here to bypass the “Comments to the Author” section, enter your conflict of interest statement in the “Confidential to Editor” section, and submit your "Accept" recommendation.

Reviewer #3: All comments have been addressed

**********

2. Is the manuscript technically sound, and do the data support the conclusions?

The manuscript must describe a technically sound piece of scientific research with data that supports the conclusions. Experiments must have been conducted rigorously, with appropriate controls, replication, and sample sizes. The conclusions must be drawn appropriately based on the data presented.

Reviewer #3: Yes

**********

3. Has the statistical analysis been performed appropriately and rigorously?

Reviewer #3: Yes

**********

4. Have the authors made all data underlying the findings in their manuscript fully available?

The PLOS Data policy requires authors to make all data underlying the findings described in their manuscript fully available without restriction, with rare exception (please refer to the Data Availability Statement in the manuscript PDF file). The data should be provided as part of the manuscript or its supporting information, or deposited to a public repository. For example, in addition to summary statistics, the data points behind means, medians and variance measures should be available. If there are restrictions on publicly sharing data—e.g. participant privacy or use of data from a third party—those must be specified.

Reviewer #3: (No Response)

**********

5. Is the manuscript presented in an intelligible fashion and written in standard English?

PLOS ONE does not copyedit accepted manuscripts, so the language in submitted articles must be clear, correct, and unambiguous. Any typographical or grammatical errors should be corrected at revision, so please note any specific errors here.

Reviewer #3: Yes

**********

6. Review Comments to the Author

Please use the space provided to explain your answers to the questions above. You may also include additional comments for the author, including concerns about dual publication, research ethics, or publication ethics. (Please upload your review as an attachment if it exceeds 20,000 characters)

Reviewer #3: Common method bias – Variance accounted for by first unrotated factor (22.711%) is now reported correctly.

No placeholder remains.

Sequential relationships – A post-hoc exploratory SEM was performed and reported and the pathway was found to be Product Quality → Satisfaction → Team Identification → Loyalty, which is a pathway that is consistent with the sequential pathway.

Gender disparity – 8% sample size for females is recognized and a rationale provided (too small for multi-group SEM) and recommendation made for future research.

Citation error – The reference with the future date (Akgül et al., 2026) is deleted.

The Recommendation for Replication – Across Multiple CSL Venues and Seasons has been included in the Conclusion.

**********

7. PLOS authors have the option to publish the peer review history of their article (what does this mean?). If published, this will include your full peer review and any attached files.

If you choose “no”, your identity will remain anonymous but your review may still be made public.

**Do you want your identity to be public for this peer review?** For information about this choice, including consent withdrawal, please see our Privacy Policy.

Reviewer #3: No

**********

# Editor report: Acceptance letter

**DOI:** 10.1371/journal.pone.0350647.r006

**Authors:** Clementswami Sukumaran

PONE-D-26-07142R2

PLOS One

Dear Dr. Tian,

I'm pleased to inform you that your manuscript has been deemed suitable for publication in PLOS One. Congratulations! Your manuscript is now being handed over to our production team.

At this stage, our production department will prepare your paper for publication. This includes ensuring the following:

* All references, tables, and figures are properly cited

* All relevant supporting information is included in the manuscript submission,

* There are no issues that prevent the paper from being properly typeset

You will receive further instructions from the production team, including instructions on how to review your proof when it is ready. Please keep in mind that we are working through a large volume of accepted articles, so please give us a few days to review your paper and let you know the next and final steps.

Lastly, if your institution or institutions have a press office, please let them know about your upcoming paper now to help maximize its impact. If they'll be preparing press materials, please inform our press team within the next 48 hours. Your manuscript will remain under strict press embargo until 2 pm Eastern Time on the date of publication. For more information, please contact onepress@plos.org.

You will receive an invoice from PLOS for your publication fee after your manuscript has reached the completed accept phase. If you receive an email requesting payment before acceptance or for any other service, this may be a phishing scheme. Learn how to identify phishing emails and protect your accounts at https://explore.plos.org/phishing.

If we can help with anything else, please email us at customercare@plos.org.

Thank you for submitting your work to PLOS ONE and supporting open access.

Kind regards,

PLOS ONE Editorial Office Staff

on behalf of

Dr. Clementswami Sukumaran

Academic Editor

PLOS One
